# From Farm to Table: Follow-Up of Shiga Toxin-Producing *Escherichia coli* Throughout the Pork Production Chain in Argentina

**DOI:** 10.3389/fmicb.2016.00093

**Published:** 2016-02-08

**Authors:** Rocío Colello, María E. Cáceres, María J. Ruiz, Marcelo Sanz, Analía I. Etcheverría, Nora L. Padola

**Affiliations:** Laboratorio de Inmunoquímica y Biotecnología, Centro de Investigación Veterinaria de Tandil – Consejo Nacional de Investigaciones Científicas y Técnicas – Comisión de Investigaciones Científicas de la Provincia de Buenos Aires, Facultad de Ciencias Veterinarias, Universidad Nacional del Centro de la Provincia de Buenos AiresTandil, Argentina

**Keywords:** STEC, foodborne pathogens, pork production chain, prevalence, characterization

## Abstract

Pigs are important reservoirs of Shiga toxin-producing *Escherichia coli* (STEC). The entrance of these strains into the food chain implies a risk to consumers because of the severity of hemolytic uremic syndrome. This study reports the prevalence and characterization of STEC throughout the pork production chain. From 764 samples, 31 (4.05%) were *stx* positive by PCR screening. At farms, 2.86% of samples were *stx* positive; at slaughter, 4.08% of carcasses were *stx* positive and at boning rooms, 6% of samples were *stx* positive. These percentages decreased in pork meat ready for sale at sales markets (4.59%). From positive samples, 50 isolates could be characterized. At farms 37.5% of the isolates carried *stx1/stx2* genes, 37.5% possessed stx2e and 25%, carried only stx2. At slaughter we detected 50% of isolates positive for stx2, 33% for stx2e, and 16% for stx1/stx2. At boning rooms 59% of the isolates carried stx1/stx2, 14% stx2e, and 5% stx1/stx2/stx2e. At retail markets 66% of isolates were positive for stx2, 17% stx2e, and 17% stx1/stx2. For the other virulence factors, *ehxA* and *saa* were not detected and *eae* gene was detected in 12% of the isolates. Concerning putative adhesins, *agn43* was detected in 72%, *ehaA* in 26%, *aida* in 8%, and *iha* in 6% of isolates. The strains were typed into 14 *E. coli* O groups (O1, O2, O8, O15, O20, O35, O69, O78, O91, O121, O138, O142, O157, O180) and 10 H groups (H9, H10, H16, H21, H26, H29, H30, H32, H45, H46). This study reports the prevalence and characterization of STEC strains through the chain pork suggesting the vertical transmission. STEC contamination originates in the farms and is transferred from pigs to carcasses in the slaughter process and increase in meat pork at boning rooms and sales markets. These results highlight the need to implement an integrated STEC control system based on good management practices on the farm and critical control point systems in the food chain.

## Introduction

Shiga toxin-producing *Escherichia coli* (STEC) are important foodborne pathogens that can cause severe disease, including a life-threatening complication such as bloody diarrhea and hemolytic uremic syndrome (HUS; Paton and Paton, 1998). HUS is one of the most common etiologies for acute kidney injury and an important cause of acquired chronic kidney disease in children (Grisaru, 2014). This damage is produced by the action of cytotoxins Stx1 and Stx2, being Stx2 and their subtypes associated more frequently with HUS (Beutin et al., 2007). The ability to adhere to epithelial cells is an important virulence trait, because adherence presumably enables to deliver toxins efficiently to host organs (Tarr et al., 2000). Intimin, encoded by *eae* gene, is required for intimate bacterial adhesion to epithelial cells inducing a characteristic histopathological lesion defined as “attaching and effacing” (A/E) and has been considered as a risk factor for disease in human (Ethelberg et al., 2004). However, the presence of *eae* would not be essential for pathogenesis, considering that some *eae* negative STEC have been associated with severe disease in human (Paton et al., 2001). Some studies reported adherence factors other than intimin, such as Saa (Paton et al., 2001), AIDA and Agn43 (Restieri et al., 2007), EhaA (Wu et al., 2010), Iha (Tarr et al., 2000; Szalo et al., 2002). AIDA was identified in diffusely adhering diarrheagenic *E. coli* strain and is associated with edema disease and diarrhea in pigs (Niewerth et al., 2001), contributing to bacterial intercellular aggregation and biofilm formation (Restieri et al., 2007); *iha* encode for an outer membrane protein identified as a bacterial adherence conferring iron regulated gene (Tarr et al., 2000) and Agn43 and EhaA are autotransporter proteins of O157:H7 involved in adhesion and biofilm formation (Wells et al., 2008). Other factors are also involved in human pathogenicity such as a plasmid that encoded enterohemolysin (EhxA), among others (Feng and Reddy, 2013).

Argentina, where the HUS is endemic, hold the highest record worldwide of this syndrome with an incidence of 17/100,000 children less than 5 years old (Rivas et al., 2010). Although STEC O157:H7 is recognized as the most important serotype associated with human infection, there are more than 400 non-O157 serotypes that have been involved in human disease and isolated from different reservoirs including cattle, pigs, goats, sheep, cats, and dogs (Parma et al., 2000; Padola et al., 2004; Amézquita-López et al., 2014). STEC usually do not produce disease in animals, however, the Stx2e subtype is involved in edema disease in pigs, a peracute toxemia characterized by vascular necrosis, edema, neurological signs and that in some cases can be fatal (Niewerth et al., 2001). STEC strains have been isolated from pork products and have been associated with human infections as diarrhea and HUS, including strains harboring *stx2e* subtype (Sonntag et al., 2005; Kaufmann et al., 2006; Trotz-Williams et al., 2012); however, it is unknown if the contamination of pork- derivate food occurs during the processing or by cross contamination (Tseng et al., 2014).

There is an increase in worldwide demand for fast-growing species with efficient feed conversion rates, such as pigs, because they represent a major share in the growth in the livestock subsector (Food and Agriculture Organization [FAO], 2014). Because of the limited epidemiologic data of STEC in pork and the increasing role of non-O157 STEC in human illnesses, it is very important to study the role of pigs as reservoirs of STEC and the transmission to the swine production chain (Ercoli et al., 2015). Taking into account the data mentioned above, the aim of this study was to determine the prevalence and to characterize STEC throughout the pork production chain in Argentina.

## Materials and Methods

### Management of Farms and Animals

The study was conducted in two pig production farm systems. Both farms are intensively organized in total confinement. The production stages are: gestation, farrowing, weaning, and growing/finishing (fattening), which are geographically separated from each other within the same farm. The usual group size varies between 10 and 30 pigs. Pigs and employees move from one building to others by corridors that are isolated from external traffic.

### Management of Carcasses Until Retails Markets

Pigs at finishing stage are transported to the slaughterhouses. After slaughtered, the pork carcasses are chilled during 24 to 48 h and sent to boning rooms in refrigerated trucks.

At the boning rooms the carcasses were boning to products such as meat and minced meat. Finally, the products are transferred to the retails markets.

### Collection of Samples

Seven hundred and sixty four samples were collected from May, 2012 to November, 2014 from two pig production systems.

This study was carried out in accordance with the recommendations of the Animal Welfare Committee from the Veterinary Science Faculty, UNCPBA, Resolution 087/02.

### Samples at Farms

Three hundred and forty eight samples were taken at farms. From these, 277 corresponded to rectal swabs, and 71 come from the environment obtained from water drink, feed and feces on the floor by swabbing.

### Samples at Slaughterhouses

One hundred and forty seven samples were by swabbing. Off these, 22 were from rectal swabs after slaughter, 85 from carcasses, and 40 from the slaughterhouses environment (pre-washing, scalding, deharing, dressing, cooling, and knives).

**FIGURE 1:**
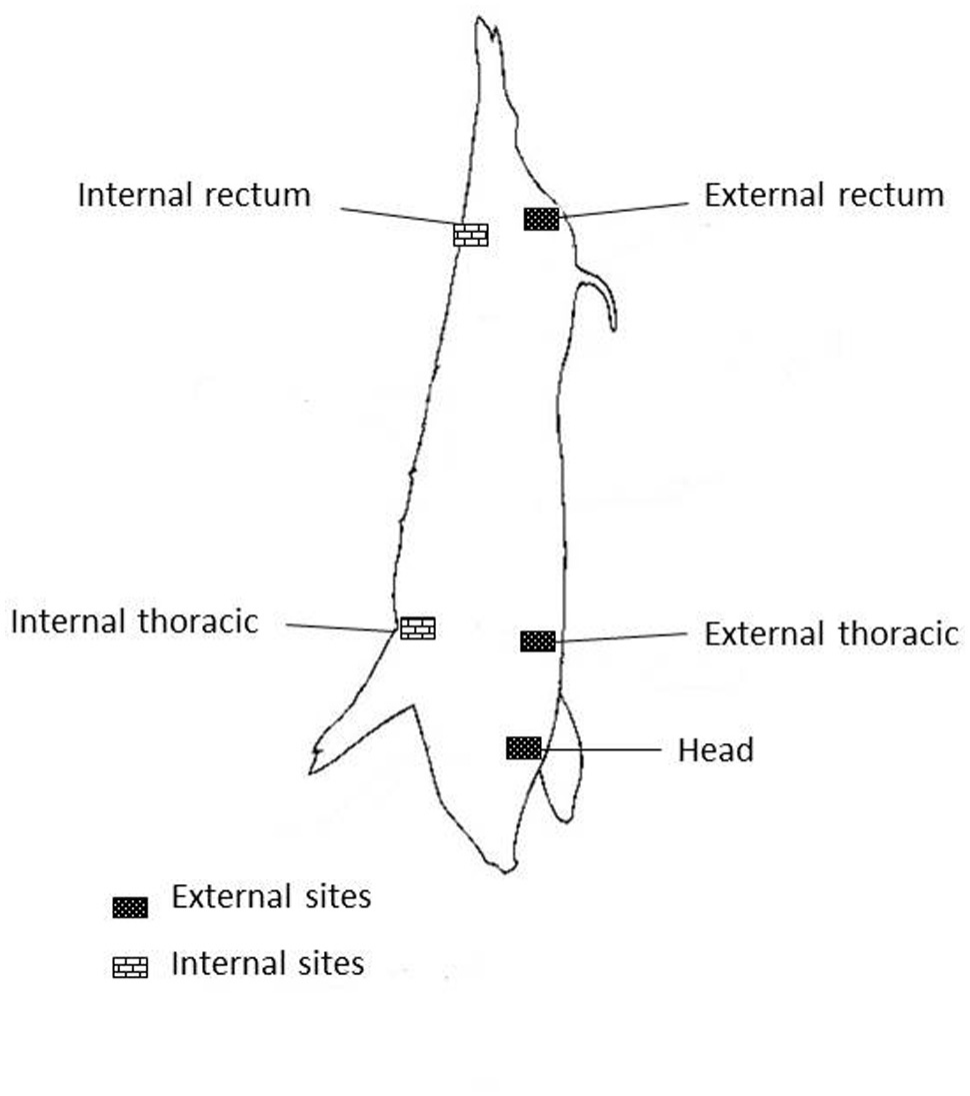
**Sites on pig carcass**.

Carcasses swabs were taken in concordance with circular No 3496/02 of Servicio Nacional de Sanidad y Calidad Agroalimentaria (SENASA, 2002). Five quarters areas of 100 cm^2^ each one were taken and processed separately, they are named heads (H), external rectum (ER), internal rectum (IR), external thoracic (ET), and internal thoracic (IT; **Figure 1**).

### Samples at Boning Rooms

One hundred and eighty one samples were taken. From these, 94 come from carcasses, 24 from meat, 23 from minced meat, and 40 from environmental samples (refrigerated trucks and meat contact surfaces such as meat tables, knives, meat mincing machine, and vertical band saw machine).

### Sampling at Retail Markets

Eighty seven samples were taken from retail markets (43 samples come from meat, 13 from minced meat, and 31 from the environment). The environmental samples were obtained from meat tables, knives, vertical band saw machine, and refrigerators.

### Sample Preparation and Isolation of STEC

Swabs were processed according to Etcheverría et al. (2010). Briefly, the swabs were cultured in Luria Bertani broth (LB) with shaking at 37°C for 18 h, and then an aliquot was grown on MacConkey agar plates by incubating at 37°C for 24 h.

Ten to fifty individual colonies were processed for amplification of Shiga toxin genes (*stx1*, *stx2*, and *stx2e*; **Table 1**). Each positive colony for either *stx* was tested for the presence of the *eae*, *ehxA*, and *saa* by multiplex polymerase chain reaction (PCR; Paton and Paton, 2002). Genes encoding adhesins (*ehaA, agn43, iha*, *aida*) were amplified using monoplex PCR. STEC strains used as positive control were *E. coli* O157:H7 EDL933 (*stx1*, *stx2*, *eae*, *ehxA*, *ehaA, agn43, iha*), *E. coli* O8 (stx2e), *E. coli* O91:H21 (*stx_1_, stx_2_, ehxA, saa*), and *E. coli* O157:H19 (*aida*).

**Table 1:** Genes, primers sequences, and size of amplified product of Shiga toxin-producing *Escherichia coli* (STEC).

Gene	Primers sequences (5′-3 ′)	Size of amplified product (bp)	Reference
*stx1*	stx1F-ATAAATCGCCATTCGTTGACTA	180	Paton and Paton, 1998
	stx1R-AGAACGCCCACTGAGATCATC			
*stx2*	stx2F-GGCACTGTCTGAAACTGCTCC	255	Paton and Paton, 1998
	stx2R-TCGCCAGTTATCTGACATTCTG			
*stx2e*	vt23F- CCGTCAGGACTGTCTGAAAC	663	Woodward et al., 1992
	vt24R- GGACGCGATAATTAAACCG			
*eae*	eaeF-GACCCGGCACAAGCATAAGC	384	Paton and Paton, 1998
	eaeR-CCACCTGCAGCAACA-AGAGG			
*ehxA*	hlyAF-GCATCATCAAGCGTACGTTCC	534	Paton and Paton, 1998
	hlyAR-AATGAGCCAAGCTGGTTAAGCT			
*saa*	saaDF-CGTGATGAACAGGCTATTGC-	119	Paton and Paton, 1998
	saaDR-ATGGACATGCCTGTGGCAAC			
*agn43*	agn43F-CTGGAAACCGGTCTGCCCTT	433	Restieri et al., 2007
	agn43R-CCTGAACGCCCAGGGTGATA			
*aida*	aida1FACAGTATCATATGGAGCCACTC	587	Restieri et al., 2007
	aida1R-TGTGCGCCAGAACTATTAATGG			
*ehaA*	ehaAF-AGGCATGAGACACGATC	500	Wu et al., 2010
	ehaAR-d AAGTCGTGCCATTGAGC			
*iha*	ihaF-CAAATGGCTCTCTTCCGTCAATGC	925	Szalo et al., 2002
	ihaR-CAGGTCGGGGTTACCAAGT			


Amplification products were separated by electrophoresis on 2% agarose gel containing 0.8 μg/ml of ethidium bromide in running buffer and were visualized by UV transillumination.

### Determination of Serotype

O and H types were determined by microagglutination technique in plates and tubes as described by Guinée et al. (1981) and modified by Blanco et al. (1996) using all available O (O1–O175) antisera plus six putative new O antigens (OX176 through OX181) and H (H1–H56) antisera (Pradel et al., 2000).

## Results

The results indicate that STEC occurrence is widespread throughout pork production chain. Among the 764 samples, 31 (4.05%) were positive for *stx*. In rectal swabs from the different pig categories, 2.86% were STEC positive, distributed 5.88% at fattening, 4.3% at growing, 2.38% at gestation and 1.51% at farrowing. STEC were not detected in feed, water, and fecal samples taken from farms. At slaughter, 4.08% of carcasses sampled were *stx* positive. The distribution in the different quarters of the carcasses was: 50% from ER, 16.6% from ET, 16.6% from IT, 16.6 % from heads. At boning rooms, 6% of samples were STEC positive, belonging 82% to carcasses, and 18% to pork meat. The distribution in the different quarters of the carcasses was: 33.3% from ER, 22.2% from IR, 22.2% from IT, 11.2% from ET and 11% from head. At sale markets 4.59% of STEC positive samples were detected in pork meat ready for sale.

### Characterization of STEC

From positive samples, 50 isolates could be characterized by PCR. In samples from farms 6/16 (37.5%) of the isolates carried *stx1*/*stx2*, 6/16 (37.5%) possessed *stx2e*, and 4/16 (25%) carried *stx2*. At slaughter 3/6 (50%) of isolates were positive for *stx2*, 2/6 (33%) for *stx2e*, and 1/6 (16%) for *stx1/stx2*. At boning rooms 13/22 (59%) of the isolates carried *stx1*/*stx2*, 3/22 (14%) *stx2e*, and 1/22 (5%) *stx1*/*stx2*/*stx2e*. At retail markets 4/6 (66%) of isolates were positive for *stx2*, 1/6 (17%) for *stx2e*, and 1/6 (17%) for *stx1/stx2*. Other virulence factors such as *ehxA* and *saa* were not detected and *eae* was detected in 6/50 (12%) of samples. Concerning putatives adhesins, *agn43* was detected in 36/50 (72%), *ehaA* in 13/50 (26%), *aida* in 4/50 (8%), and *iha* in 3/50 (6%) of isolates. The most frequent virulence profiles found were *stx1, stx2*, or *stx2e* combined with *agn43* in 36 (72%) strains. The 50 isolates were typed into 14 *E. coli* O groups (O1, O2, O8, O15, O20, O35, O69, O78, O91, O121, O138, O142, O157, O180) and 15 were considered O non-typable (NT). Ten H antigens (H9, H10, H16, H21, H26, H29, H30, H32, H45, H46) were distributed among the 50 strains, while one isolate were non-motile (H–). **Table 2** indicates the relationships between virulence profiles, sites of samples and serotypes in isolated STEC strains.

**Table 2:** Relationships between virulence profiles, sites of samples, and serotypes in STEC strains.

Sample site	Serotypes	No. of strains	Virulence profile
**Farms (pigs)**			
Gestation	O1:HNM	1	*stx1*
Gestation	O1:HNM	1	*stx1,stx2, agn43, ehaA*
Fattering	O2:H32	3	*stx2, agn43*
Growing and Farrowing	O8:H9	4	*stx2e, agn43*
Fattering	O20:H9	1	*stx2, agn43*
Fattering	O142:H26	1	*stx2*
Fattering	ONT:H16	1	*stx2e, agn43, iha*
Fattering	ONT:H26	1	*stx1,stx2, agn43*
Fattering	ONT:H32	1	*stx2e, agn43, ehaA*
Growing	ONT:HNM	1	*stx2e, agn43*
Fattering	ONT:HNM	1	*stx1,stx2, agn43*
**Slaugtherhouse**		
H	O1:H9	1	*st2xe, ehaA*
ER	O91:H21	1	*stx1, agn43, ehaA, iha*
ER	ONT:H29	1	*stx1,stx2*
ER	ONT:HNM	1	*stx2*
ET and IT	O8:HNM	2	*stx2e, ehaA*
**Boning rooms**			
ET	O8:HNM	1	*stx2*
ER	O15:H45	2	*stx1,stx2,ehaA*
IR	O35:H10	1	*stx1,stx2, agn43*
ET	O69:HNM	1	*stx1,stx2, agn43*
H	O78:H45	3	*stx1,stx2, agn43*
H	O78:HNM	2	*stx1,stx2,agn43*
ET	O121:H21	1	*stx2e*
ET	O138:H30	1	*stx2e*
IT and meat	O157:H21	4	*stx2, eae, aida, agn43, ehaA*
ET	O180:H21	1	*stx1,stx2*
ET	ONT:H21	1	*stx1,stx2*
Meat	ONT:H21	2	*stx2e, agn43*
IR	ONT:H32	1	*stx1,stx2, agn43*
IT	ONT:HNM	1	*stx2, stx2e, eae, agn43*
**Sale markets**			
Meat	O9:H21	1	*stx2, agn43*
Meat	O9:HNM	2	*stx2, agn43*
Meat	ONT:H21	1	*stx2e, agn43*
Meat	ONT:HNM	1	*stx2, sxt2e, agn43*
meat	ONT:HNM	1	*stx2, stx2e, eae, agn43*


## Discussion

To our knowledge, this study is the first that reports the prevalence and characterization of STEC strains through the chain pork suggesting the vertical transmission of these pathogens. However, there are studies that demonstrate the prevalence in farms, finishing pigs, slaughter, and pork meat in sales markets, separately. The prevalence of STEC in pigs, carcasses, and pork meat at different stage of production from other countries is variable and it is necessary to take caution when comparing prevalence since the variation may be due to several factors, such as sampling method, samples processing, and season in which the study was performed. Different prevalence of STEC in pigs was reported previously, ranged from 2 to 31%, in agreement with our results (Parma et al., 2000; Kaufmann et al., 2006; Meng et al., 2014). In carcasses at slaughter and boning rooms, the prevalence found is in concordance with other studies whose prevalence ranged from 0.2 to 26% (Leung et al., 2001; Bouvet et al., 2002; Bohaychuk et al., 2011; Koláčková et al., 2014).

At slaughter some operations such as skinning, evisceration and handling are more likely than others to contaminate carcass and meat (Koohmaraie et al., 2005; Etcheverría et al., 2010). For this, some areas of carcasses are more prone than others to be exposure to potential or cross contamination, thus the suggestion of sampling at three or four sites on carcass, because contamination appears to vary considerably among different sites (Roberts et al., 1984). In addition, the ER is the area that involves a particular risk of contamination during early stages of dressing as our result in that the ER was the more contaminated area, in concordance with Bouvet et al. (2002). However, other areas sampled as IT and IR shown more contamination probably due to handling at the boning room.

In our study, the prevalence at sale market was less than that informed by Magwedere et al. (2013) in USA (50%), Martin and Beutin (2011) in Germany (14%) and Lee et al. (2009) Korea (15%). This could be due because these studies were performed in sale markets where meat from different origins were sold and cross contamination during handling can occur. In the present study the samples were obtained from sale markets where only meat pork was sold.

From 50 STEC isolates, *stx*1/*stx*2 and *stx*2 occurred more frequently than isolates carrying *stx1.* Epidemiologically, Stx2-producing strains are more often related with HUS than strains that produce Stx1 (Paton and Paton, 2002).

Regarding *stx*2e, its prevalence decreased from pigs at farms to pork meat. Although some authors have reported the presence of *stx2*e in STEC strains in human patients on a few occasions, STEC harboring *stx2*e are more likely to cause edema disease in pigs causing economic losses in pig production (Kaufmann et al., 2006). It is necessary to determine a rol of these strains in human infection.

The presence of *eae* detected in isolates that harbor too *stx2*, *stx2e, agn43* from boning rooms and sale markets implies a high risk for human health (Tseng et al., 2014). The most prevalent adhesin identified among all isolates and involved in adhesion and biofilm formation was Agn43, followed by EhaA in agreement with Biscola et al. (2011) and Tseng et al. (2014) which detected them in swine and different sources, respectively. In this study, 8% of strain harbored *AIDA*, similar to that found in South Africa (Mohlatlole et al., 2013) and China (Zhao et al., 2009). The *iha* was present in few isolates, but this gene has been detected over 70% of the *eae*-negative STEC strains associated with human clinical cases examined in studies in Germany (Hauser et al., 2013) and Argentina (Galli et al., 2010). The high prevalence of LEE negative STEC isolated from pigs in our study emphasizes the need of further work to better define the role that the attachment proteins outside the LEE may play in the adherence to both pork and human epithelial cells.

Although many serotypes isolated in this study have been detected with low incidence in human disease and rarely associated with outbreaks (Friedrich et al., 2002), they have been isolated from pigs, sheep, cattle and food in other countries (Beutin et al., 1993; Kaufmann et al., 2006; Beutin et al., 2007). At slaughter and boning rooms serogroups associated with human illnesses such as *E. coli* O91, O121, and O157 were detected in agreement with other studies that recovered these serogroups from pig fecal samples (Desrosiers et al., 2001; Friedrich et al., 2002; Karmali et al., 2003; Bielaszewska et al., 2009; Trotz-Williams et al., 2012; Yoon et al., 2013; Tseng et al., 2014).

## Conclusion

The presented study investigated the tracking of STEC from the farm to table and indicates that the production of meat pork harbored STEC strains. STEC contamination originated in the farms is transferred from pigs to carcasses in the slaughter process and increase in meat pork at boning rooms and sales markets. Besides, the entrance of these strains into the food chain implies a risk to consumers because of severity of the illness they can cause. If STEC is present in any food product, it has the possibility of causing foodborne illness. In addition to public health problem, the presence of strains carrying the *stx2e* gene is a problem for the pig production because they can cause the edema disease causing important economic losses. In spite of the wealth of data available on this important disease, it is necessary to effectively prevent this contamination by educating employees, retailers and consumers on the appropriate handling and storage of meat. Further studies are needed to provide more systematic data in order to fuel the development of novel approaches for control of STEC in foods, including pork meat.

## Author Contributions

RC conceived, designed, analyzed the experiments, and wrote the manuscript. MR, MS, and MC did some of the experiments. NP and AE designed some of the experiments, analyzed the data, and revised the manuscript.

## Conflict of Interest Statement

The authors declare that the research was conducted in the absence of any commercial or financial relationships that could be construed as a potential conflict of interest.
